# Efficiently Specified Ventral Midbrain Dopamine Neurons from Human Pluripotent Stem Cells Under Xeno‐Free Conditions Restore Motor Deficits in Parkinsonian Rodents

**DOI:** 10.5966/sctm.2016-0073

**Published:** 2016-10-14

**Authors:** Jonathan C. Niclis, Carlos W. Gantner, Walaa F. Alsanie, Stuart J. McDougall, Chris R. Bye, Andrew G. Elefanty, Edouard G. Stanley, John M. Haynes, Colin W. Pouton, Lachlan H. Thompson, Clare L. Parish

**Affiliations:** ^1^ The Florey Institute of Neuroscience and Mental Health, University of Melbourne, Melbourne, Victoria, Australia; ^2^ Murdoch Children’s Research Institute, The Royal Children’s Hospital, Melbourne, Victoria, Australia; ^3^ Department of Anatomy and Developmental Biology, Monash University, Clayton, Victoria, Australia; ^4^ Department of Paediatrics, University of Melbourne, Melbourne, Victoria, Australia; ^5^ Monash Institute of Pharmaceutical Sciences, Monash University, Clayton, Victoria, Australia

**Keywords:** Human pluripotent stem cells, Ventral midbrain, Dopamine neurons, Parkinson’s disease, Xenogeneic‐free, LMX1A, PITX3

## Abstract

Recent studies have shown evidence for the functional integration of human pluripotent stem cell (hPSC)‐derived ventral midbrain dopamine (vmDA) neurons in animal models of Parkinson’s disease. Although these cells present a sustainable alternative to fetal mesencephalic grafts, a number of hurdles require attention prior to clinical translation. These include the persistent use of xenogeneic reagents and challenges associated with scalability and storage of differentiated cells. In this study, we describe the first fully defined feeder‐ and xenogeneic‐free protocol for the generation of vmDA neurons from hPSCs and utilize two novel reporter knock‐in lines (LMX1A‐eGFP and PITX3‐eGFP) for in‐depth in vitro and in vivo tracking. Across multiple embryonic and induced hPSC lines, this “next generation” protocol consistently increases both the yield and proportion of vmDA neural progenitors (OTX2/FOXA2/LMX1A) and neurons (FOXA2/TH/PITX3) that display classical vmDA metabolic and electrophysiological properties. We identify the mechanism underlying these improvements and demonstrate clinical applicability with the first report of scalability and cryopreservation of bona fide vmDA progenitors at a time amenable to transplantation. Finally, transplantation of xeno‐free vmDA progenitors from LMX1A‐ and PITX3‐eGFP reporter lines into Parkinsonian rodents demonstrates improved engraftment outcomes and restoration of motor deficits. These findings provide important and necessary advancements for the translation of hPSC‐derived neurons into the clinic. Stem Cells Translational Medicine *2017;6:937–948*

**Significance Statement**

The authors report the generation of highly pure midbrain dopamine cultures under feeder‐free, fully defined, and xeno‐free conditions from human pluripotent stem cells. Xeno‐free differentiated cells display gene, protein, and electrophysiological properties of midbrain neurons, as well as improved grafting outcomes in Parkinsonian rodents, observations enhanced by the use of two novel reporter lines of interest to this research field. Furthermore, for the first time, ventral midbrain dopamine neurons were amenable to scalability and cryopreservation, crucial steps for the advancement of cell replacement therapy in Parkinson’s disease.

## Introduction

Clinical trials of human fetal tissue grafts in Parkinson’s disease patients provide evidence that donor neural cells can reinnervate appropriate targets, increase dopamine neurotransmission, and reverse phenotypic symptoms for decades after transplantation [1]. However, immunological rejection as well as temporal and cellular heterogeneity of scarce fetal tissue has resulted in variable symptomatic relief [2]. Human pluripotent stem cells (hPSCs) have the potential to circumvent these obstacles, presenting an unrivalled regenerative medicine tool for brain repair. Unlike extensive earlier works, only in recent years have protocols emerged that are capable of generating bona fide ventral midbrain dopamine (vmDA) neurons from hPSCs, with success largely attributed to early floor‐plate patterning [3, 4, 5, 6]. Within these studies vmDA neurons were definitively identified using coexpression of key markers. Broadly, ventricular zone equivalent progenitors were confirmed using combinatorial assessment of forebrain/midbrain marker OTX2, basal plate marker FOXA2 and floor plate marker LMX1A expression. Intermediate zone equivalent progenitors were distinguished by FOXA2 coexpression with midbrain identifiers NURR1 or engrailed‐1 (EN1). Finally, mantle zone neurons were confirmed by colocalization of FOXA2 and the dopamine (DA) marker tyrosine hydroxylase (TH) and in some cases the cardinal vmDA neuronal identifier PITX3. Recent grafting studies of these hPSC‐derived DA precursors demonstrated their capacity to survive, partially innervate the striatum of 6‐hydroxydopamine (6‐OHDA) lesioned rodents or primates, and ameliorate motor function via neuronal integration [4, 5, 7, 8, 9].

Although these new protocols are more sophisticated than early methods that generated non‐region‐specific DA neurons [10, 11], they retain critical components that impede clinical compliance requirements of regulatory authorities. Specifically, all protocols utilize hPSCs adapted to mouse embryonic fibroblast (MEF) coculture, and in several protocols MEF coculture or conditioned media is required immediately preceding or during initiation of neural induction [4, 6, 12]. MEFs impart immunogenic proteins, pluripotency promoting growth factors, and extracellular matrix proteins [13, 14], as well as exhibiting batch‐to‐batch stochasticity, which together affect hPSC growth, morphology, and differentiation [15]. Although some steps have been taken to move toward a clinically translatable protocol [16], all protocols retain poorly defined xenogeneic factors throughout induction and maturation stages, including extracellular matrices such as Engelbreth‐Holm‐Swarm sarcoma‐derived Matrigel (Thermo Fisher Scientific, Waltham, MA, https://www.thermofisher.com) or laminin, B27 supplement, and knockout serum replacement (KSR) media [3, 4, 5, 6, 7, 8, 12].

Cell replacement therapy requires elimination of xenogeneic components from hPSC expansion and differentiation protocols. To date these changes have not been fully implemented and comparisons have not been made to prior published xenogeneic differentiation protocols. In this study, we describe a fully defined feeder‐ and xeno‐free protocol for the generation of bona fide vmDA neurons. Currently, the dual‐SMAD inhibition system reports the highest proportions of correctly patterned vmDA lineage cells, as assessed by colocalization of relevant vmDA proteins [4] and consequently was utilized as a comparative benchmark. Investigation of the newly developed xeno‐free system, utilizing novel LMX1A‐eGFP and PITX3‐eGFP knock‐in reporter lines, demonstrates not only feasibility but also significant improvement in ventral midbrain (vm) patterning, yield, and population homogeneity. Generated DA neurons exhibit characteristic vmDA electrophysiological properties and DA metabolism. Moreover, xeno‐free vmDA progenitors resulted in improved phenotype specification upon transplantation into Parkinsonian rodents and were shown for the first time to restore functional motor deficits. Finally, we demonstrate that this “next generation” protocol is readily scalable and for the first time report successful cryopreservation of hPSC‐derived vmDA progenitors at a time amenable to transplantation. These findings provide critical advancements for the generation and utility of hPSC‐derived vmDA neurons for in vitro purposes such as disease modeling and drug development and importantly for clinical transplantation in Parkinson’s disease patients.

## Materials and Methods

### Maintenance of Human Pluripotent Stem Cells

Human embryonic stem cell (hESC) lines HES3 [17], H9 (WA‐09; WiCell [18]), our newly generated H9 reporter line (LMX1A‐eGFP knock‐in), recently generated PITX3‐eGFP knock‐in [19], and integration‐free human induced pluripotent stem cell (hiPSC) lines RM3.5 (A.G.E. and E.G.S., unpublished results) and 409B2 [20], were cultured in xenogeneic or xeno‐free conditions. All lines were confirmed to be karotypically normal and frequently tested for absence of mycoplasma (MycoAlert detection kit; Lonza, Basel, Switzerland, http://www.lonza.com). Xenogeneic conditions: hPSCs were cocultured on 0.02 × 10^6^/cm^2^ γ‐irradiated MEFs in Dulbecco’s modified Eagle’s medium (DMEM)/F12 + GlutaMAX (Thermo Fisher Scientific) with 20% KSR (Thermo Fisher Scientific), 1% NEAA (Thermo Fisher Scientific), 0.2% β‐mercaptoethanol (Thermo Fisher Scientific), 0.4% penicillin‐streptomycin (Thermo Fisher Scientific), and 10 ng/ml FGF2 (R&D Systems, Minneapolis, MN, https://www.rndsystems.com). Xeno‐free conditions: hPSCs were cultured on laminin‐521 (Biolamina, Matawan, NJ, http://www.biolamina.com), in xeno‐free TeSR2 (StemCell Technologies, Vancouver, BC, Canada, https://www.stemcell.com) supplemented with 0.4% penicillin‐streptomycin. hPSC lines were adapted to the TeSR2 xeno‐free conditions and maintained for >20 passages. Media were changed daily, and EDTA was used to passage the cells every 4–6 days. Cultures were maintained at 37°C with 5% CO_2_.

### vm Dopaminergic Differentiation

Confluent hPSC cultures were disassociated with EDTA to generate small aggregates. Xenogeneic hPSCs underwent MEF‐depletion before differentiation (plating onto gelatin‐coated dishes for 15 minutes in feeder conditioned media that was generated by incubating hPSC media with MEFs for 24 hours and 10 ng/ml FGF2 added before use), resulting in preferential attachment of MEFs but not hPSCs. Pluripotent cells were collected in feeder‐conditioned media and seeded at either standard (0.225 × 10^6^/cm^2^) or high densities (0.675 × 10^6^/cm^2^) on tissue culture grade plastic coated with Matrigel. For xeno‐free conditions, hPSC suspensions were collected in TeSR2 media and plated at the above densities on plates coated with laminin‐521 (10 μg/ml) or vitronectin (10 μg/ml; StemCell Technologies).

**Figure 1:**
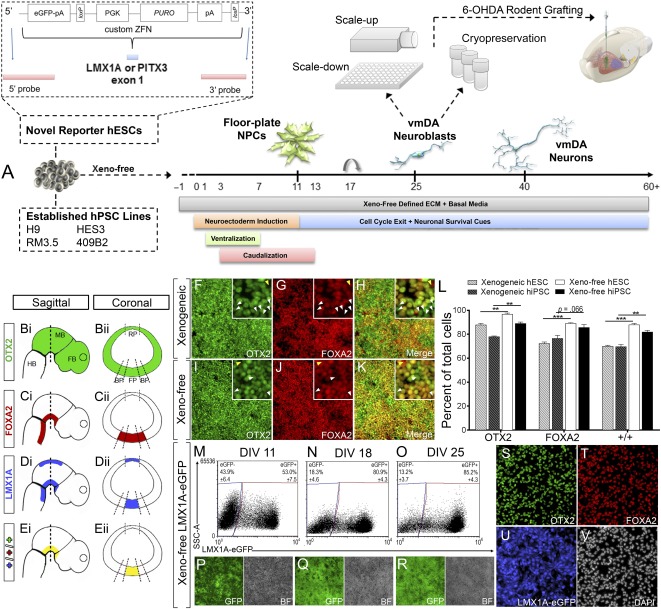
Xeno‐free vmDA protocol differentiation to enriched ventral floor plate precursors interrogated with LMX1A‐eGFP reporter line. **(A):** Study overview detailing novel reporter hPSC lines, fully defined, feeder‐free, xeno‐free differentiation protocol that is scalable and cryopreservable. **(B–E):** Developing embryo schematic illustrating sagittal **(Bi, Ci, Di, Ei)** and coronal **(Bii, Cii, Dii, Eii)** expression of key transcription factors OTX2 **(B)**, FOXA2 **(C)**, LMX1A **(D)**, and merged **(E)**, that in combination are indicative of vmDA precursors. Expression of OTX2, FOXA2, and merged at D11 under xenogeneic differentiation **(F–H)** and xeno‐free **(I–K)** protocols. Insets show higher magnification, highlighting the presence of poorly specified progenitors that express OTX2 (white arrows) or FOXA2 only (yellow arrows). **(L):** Quantification of vmDA precursors cultured under xenogeneic and xeno‐free conditions for hESCs and hiPSCs. Differentiation of LMX1A‐eGFP hESC under xeno‐free conditions confirmed correct specification of vmDA precursors with increasing expression of LMX1A from D11 to D25 by live cell flow cytometry **(M–O)** and live imaging **(P–R)**. **(S–V):** Immunostaining at D18 of OTX2 **(S)**, FOXA2 **(T)**, LMX1A‐eGFP **(U)**, and DAPI **(V)**. *n* = 3 technical and culture replicates, mean ± SEM. ∗∗, *p* < .01, ∗∗∗, *p* < .001. Immunofluorescence images are at ×100 magnification. Abbreviations: BP, basal plate; D, day; DAPI, 4′,6‐diamidino‐2‐phenylindole; FB: forebrain; FP, floor plate; GFP, green fluorescent protein; HB, hindbrain; hESC, human embryonic stem cell; hiPSC, human induced pluripotent stem cell; hPSC, human pluripotent stem cell; MB: midbrain, NPC, neural progenitor cell; vmDA, ventral midbrain dopaminergic.

Differentiation to vmDA neurons was performed as described previously [4] with modifications (Fig. 1O). Specifically, differentiation commenced 24 hours after seeding with the addition of serum replacement media (SRM; DMEM/F12+ GlutaMAX with 15% KSR [either xenogeneic or xeno‐free variant], 1% NEAA, and 0.2% β‐mercaptoethanol) and supplemented with SMAD inhibitors LDN193189 (LDN; 100 nM or 200 nM; Stemgent, Cambridge, MA, https://www.stemgent.com), and SB431542 (SB; 10 μM; R&D Systems) for neural induction. SB and LDN were removed at day (D) 5 and D11, respectively. SRM media were gradually replaced with N2 media (DMEM/F12 with 1% N2 and 1% ITS‐A [Thermo Fisher Scientific]) from D5 to D11. Sonic hedgehog C25II (100 ng/ml; R&D Systems) and the smoothened receptor agonist purmorphamine (2 μM; Stemgent) were added from D1 to D7 to promote ventralization. GSK3β inhibitor CHIR99021 (3 μM; Stemgent) was added from D3 to D13 to promote caudalization. At D11, media were switched to NBN2B27 (a 1:1 mix of neurobasal media and DMEM/F12 + GlutaMAX, supplemented with 2% B27 (either xenogeneic or xeno‐free), 1% ITS‐A, 0.5% GlutaMAX, and 0.4% penicillin‐streptomycin) with recombinant human brain‐derived neurotrophic factor (20 ng/ml; R&D Systems), recombinant human glial cell line‐derived neurotrophic factor (20 ng/ml; R&D Systems), ascorbic acid (200 nM; Sigma‐Aldrich, St. Louis, MO, http://www.sigmaaldrich.com), recombinant human transforming growth factor type β3 (TGFβ3; 1 ng/ml; PeproTech, Rocky Hill, NJ, https://www.peprotech.com), dibutyryl cAMP (0.05 mM; Tocris Bioscience, Pittsburgh, PA, https://www.tocris.com), and the notch inhibitor DAPT (10 μM, Sigma‐Aldrich), henceforth known as “maturation media.” At D17, cultures were dissociated with Accutase (Innovative Cell Technologies, Inc., San Diego, CA, http://www.accutase.com) and seeded onto surfaces coated with human laminin‐521 or poly‐L‐ornithine (15 μg/ml; Sigma‐Aldrich) and mouse laminin (3.5 μg/ml; Thermo Fisher Scientific), and fibronectin (2 μg/ml; R&D Systems). Maturation media were maintained until desired end point.

### Cryopreservation

vmDA neural progenitor cells (NPCs) were collected after 22 days of differentiation (without passage) using EDTA for 5 minutes at 37°C to generate a cell suspension composed of 10 to 200 cell clusters. Cells were resuspended in maturation media and mixed 1:1 with a xeno‐free cryopreservation solution (20% dimethyl sulfoxide, 20% TeSR2, 60% xeno‐free KSR) and immediately transferred to a slow rate freezer EF600M (Grant Instruments, Shepreth, United Kingdom, http://www2.grantinstruments.com).

### Immunocytochemistry and Cell Quantification

Cells were fixed in 4% paraformaldehyde for 7–10 minutes and antibody staining performed as previously described [17]. Images were captured using a Zeiss Axio Observer.Z1 or Zeiss Pascal Confocal Microscope. Quantification was carried out on three technical replicates/condition/experiment and repeated on at least three independent culture experiments. Statistical analysis was performed using Graphpad Prism: Student’s *t* test comparison was performed between all xenogeneic and xeno‐free conditions (*p < .05, **p < .01, ***p < .001).

### Flow Cytometry

Cells were dissociated with Accutase (4 minutes, 37°C) and stained with primary antibodies (supplemental online Table 1) according to previously described methods [21]. Appropriate unstained and single antibody controls were used to identify background fluorescence and for compensation respectively, with gating performed according to standard procedures (supplemental online Fig. 6A–6H).

### Gene Expression Analysis

Total RNA was extracted at D0, D11, D25, and D40 using Trizol. RNA was converted to cDNA and subsequently analyzed using quantitative real‐time polymerase chain reaction (qPCR) for six genes of interest (supplemental online Table 2) using previously described methods [17]. All qPCR was performed across triplicate technical replicates for each of the four independent biological replicates and normalized against HPRT1.

### High‐Performance Liquid Chromatography

Dopamine and the metabolite homovanillic acid (HVA) levels were measure in xenogeneic and xeno‐free cultures at D40 using reverse phase liquid chromatography with electrochemical detection, as previously described [16, 22]. Data were expressed as pmol/ml of DA or HVA, and dopamine turnover determined by the ratio of DA to HVA.

### Electrophysiology

Whole‐cell patch‐clamp recordings were performed in vitro on H9 PITX3‐GFP hESC‐derived DA neurons (*n* = 21) at D55–D65 using previously described methods [22]. Recording pipettes (3.5–5.5 MΩ) were filled with a low Cl‐ intracellular solution (pH 7.3 and 290 mOsmol). As a consequence, E_Cl_ = −69 mV, and inhibitory post synaptic currents (IPSCs) had negligible amplitudes at VH = −60 mV, although more prominent outward current amplitudes were achieved by shifting to VH = −40 mV. All recordings were made using a Multiclamp 700B (Molecular Devices, Sunnyvale, CA, https://www.moleculardevices.com). Signals were sampled at 20 kHz and filtered at 10 kHz (*p*‐Clamp 10.3; Molecular Devices). Voltage clamp measurements: Cells were held at −60 mV and inward spontaneous excitatory postsynaptic currents (EPSCs) were recorded for 3 minutes and blocked by AMPA receptor antagonist NBQX (20 μM; Tocris Bioscience). Cells were held at −40 mV and outward spontaneous inhibitory postsynaptic currents (sIPSCs) were recorded. Membrane potential measurements: Action potentials (APs) were stimulated in current‐clamp mode. Resting membrane potential was recorded in the course of 1 minute. Membrane potential was adjusted to −60 mV (current subtraction, −8 ± 3 pA for all cells) and cells depolarized with a series of 400‐millisecond current pulses in 10 pA increments from −40 to 160 pA. Liquid junction potentials were corrected for during data analysis (10.7 mV at 32°C) where spontaneous excitatory postsynaptic currents (sEPSCs) were detected by MiniAnalysis (Synaptosoft, Decatur, GA, http://www.synaptosoft.com) and APs counted in Clampfit (version 10.3). Biocytin (0.5%) was added to the recording pipette to fill neurons postrecording and validate DA phenotype (via TH coimmunoreactivity).

### Transplantation

All animal procedures were conducted in accordance with the Australian National Health and Medical Research Council’s published Code of Practice for the Use of Animals in Research and approved by the Florey Institute for Neuroscience animal ethics committee. Transplantation studies were performed in unilateral 6‐hydroxydopamine lesioned athymic mice and rats, as previously described [23, 24, 25]. To assess the survival and maintenance of vmDA fate specification in vivo, H9::LMX1A‐eGFP hESCs were differentiated for 25 days under xenogeneic or xeno‐free conditions. Cultures were subsequently dissociated and resuspended at 50,000 cells per μl, with 1 μl stereotaxically implanted into the denervated striatum of mice (*n* = 6 per group). Mice were killed (100 mg/kg pentobarbitone) at 5 weeks. To assess the long‐term functional integration of xeno‐free vmDA progenitors, grafts were performed into rats because of their greater responsiveness in motor behavioral tests compared with mice. Briefly, 6‐OHDA lesioned athymic rats (*n* = 15) were tested for rotational asymmetry in response to administration of d‐amphetamine sulfate (3.5 mg/kg, i.p.) 3 weeks after lesioning and retested for motor improvement at 2, 4, and 6 months after the transplantation of H9::PITX3‐eGFP derived vmDA progenitors (50,000 cells in 1 μl injected into the striatum). Rats were killed at 6 months for histological assessment.

## Results

### Establishment of a Fully Defined Feeder‐Free, Xeno‐Free vmDA Differentiation Protocol

vm differentiation cell‐seeding density was optimized at 0.675 × 10^6^ cells per cm^2^, producing uniform monolayers of tightly compacted pluripotent cells devoid of acellular regions (supplemental online Fig. 1A–Hii). hPSC lines (Fig. 1A) were then adapted from xenogeneic KSR media and MEF coculture to TeSR2 xeno‐free conditions and differentiated under fully‐defined and xeno‐free media, which resulted in widespread coexpression of the fore/midbrain marker OTX2 (Fig. 1B) and basal/floor plate marker FOXA2 (Fig. 1C) at D11 (supplemental online Fig. 1Fii, 1Gii, 1Hii) [26]. Finally, two xeno‐free matrix proteins, vitronectin and human laminin‐521, were compared for their ability to replace Matrigel (a mouse sarcoma extract). Both matrices facilitated appropriate patterning, however, only laminin‐521 supported the long‐term attachment of neural precursors (supplemental online Fig. 1I–1P). Additionally elevated levels of the small molecule bone morphogenic protein antagonist LDN‐193189 (200% higher) improved differentiation reproducibility (data not shown).

Next, a detailed assessment of differentiation outcomes in xeno‐free conditions was performed using the novel LMX1A‐ and PITX3‐eGFP reporter lines, with comparative evaluations to established xenogeneic cultures at stages corresponding to vmDA NPCs (D11–D18), neuroblasts/immature neurons (D25), and mature neurons (D40 and D60–D75; Fig. 1A).

### Xeno‐Free Conditions Generate Correctly Specified, High Purity vmDA Progenitors

Xenogeneic conditions generated OTX2 and FOXA2 expression at levels consistent with previous reports [4, 6, 7], (Fig. 1F–1H). Xeno‐free differentiation significantly increased vm NPC fate acquisition with the proportion of OTX2^+^ cells increasing (from 87.8% to 96.8% for hESC H9, and 77.9%–88.9% for hiPSC RM 3.5), as well as FOXA2^+^ cells increasing (72.2%–89.0% for hESC H9 and 76.5%–85.6% for hiPSC RM 3.5; Fig. 1I, 1J, 1L, supplemental online Fig. 2H, 2I). Xeno‐free differentiation significantly increased the proportion of OTX2/FOXA2 coexpressing cells, from 70.0% to 88.9% for hESC H9 and from 69.6% to 82.9% for hiPSC RM3.5 (Fig. 1H, 1K, 1L; supplemental online Fig. 2). Robust OTX2^+^/FOXA2^+^ homogeneity was observed across additional hPSC lines under xeno‐free conditions (91.3% of HES3 hESC and 90.3% of 409B2 hiPSCs; supplemental online Figs. 3A–3E, 4A–4E).

**Figure 2:**
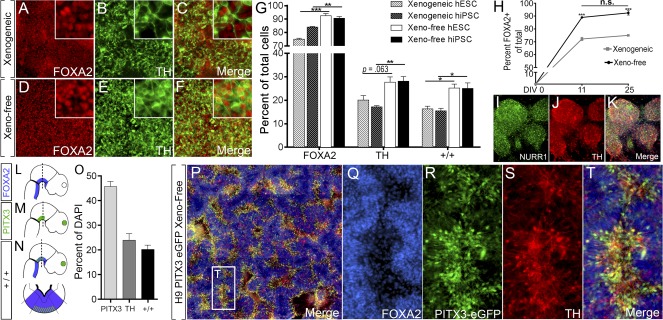
Differentiation of hPSCs under xeno‐free conditions increases generation of bona fide vmDA neurons at D25. Images of FOXA2 **(A, D)**, TH **(B, E)**, and FOXA2/TH **(C, F)** coexpression in H9 cells differentiated under xenogeneic **(A–C)** and xeno‐free **(D–F)** conditions, showing increased FOXA2/TH colabeled vmDA neurons under xeno‐free conditions. **(G):** Quantification of subpopulations. **(H):** Quantification of the temporal expression of FOXA2 reveals maintenance of early fate specification for xeno‐free conditions, yet xenogeneic cultures fail to increase FOXA2 progenitors yields through time. **(I–K):** Image of xeno‐free differentiated culture expressing NURR1 **(I)**, TH **(J)**, and merge **(K)**. **(L–N):** Sagittal schematic of the developing embryo illustrating FOXA2 **(L)**, PITX3 **(M)**, and combined FOXA2/PITX3 **(N)** expression, where vmDA neurons are born. **(O–T):** Xeno‐free differentiation and quantification of an H9::PITX3‐eGFP reporter line at D25 revealed cultures rich in bona fide vmDA neurons as revealed by FOXA2^+^/TH^+^/PITX3^+^ coexpression. *n* = 3–5 culture replicates, mean ± SEM. ∗∗, *p* < .01; ∗∗∗, *p* < .001. Immunofluorescence images are at ×100 magnification. Abbreviations: D, day; DAPI, 4′,6‐diamidino‐2‐phenylindole; hESC, human embryonic stem cell; hiPSC, human induced pluripotent stem cell; hPSC, human pluripotent stem cell; n.s., not significant; TH, tyrosine hydroxylase; vmDA, ventral midbrain dopamine.

Developmentally, LMX1A delineates the floor plate along the medial‐lateral axis of the neural tube with greater specificity than FOXA2 (Fig. 1Cii, 1Dii) [27]. Rostro‐caudally, LMX1A expression is also more restricted and indicative of the vmDA progenitor zone than FOXA2 (Fig. 1Ci, 1Di) [27]. Although LMX1A is also expressed in the dorsal neural tube, this region is FOXA2^‐^ and can therefore be excluded using coexpression stains. Assessment of OTX2 coexpression within LMX1A^+^/FOXA2^+^ cells enables further narrowing of the developmental spatial niche along the rostral‐caudal axis, with OTX2 excluding the ventral hindbrain portion of the floor plate (Fig. 1B). To date, studies of hPSC vmDA differentiation have not evaluated expression levels of this highly restricted triple positive neural progenitor (Fig. 2Ei, 2Eii). Therefore, to further characterize the specification of the vm progenitors using the xeno‐free culture system, a novel LMX1A‐eGFP knockin reporter hESC line (H9::LMX1A‐eGFP) was generated to enable tracking of these progenitors in vitro (and subsequently in vivo). This new reporter line was generated and validated using the same methodological approach to our recently derived PITX3‐GFP H9 reporter line, whereby a custom zinc finger nuclease pair in combination with a targeting vector delivered to exon 1 of the endogenous LMX1A locus an eGFP‐puromycin cassette (Fig. 1A) [19]. Using this novel reporter, live cell imaging and flow cytometric evaluation of xeno‐free cultures revealed rapid increases in eGFP with 53.0% ± 7.5% of progenitors LMX1Ae‐GFP^+^ at D11, increasing to 80.9% ± 4.3% at D18 and 85.2% ± 4.3% by D25 (Fig. 1M–1R, supplemental online Fig. 8). Cultures stained with anti‐GFP and anti‐LMX1A/B antibodies showed consistent coexpression, demonstrating the high fidelity of eGFP fluorescence in this reporter line (supplemental online Fig. 8). Furthermore, LMX1A‐eGFP^+^ quantification at D18 revealed all eGFP^+^ cells coexpressed both OTX2 and FOXA2, indicative of bona fide vmDA precursor identity (Fig. 1S‐V; supplemental online Fig. 8). LMX1A‐eGFP xenogeneic differentiation outcomes mirrored the decreased efficiencies observed from xenogenic parental H9 and iPSC controls (data not shown).

Ongoing differentiation promoted the expression of the rate‐limiting enzyme in dopamine synthesis, TH, within both xenogeneic and xeno‐free cultures by D25 (Fig. 2A–2F). Greater uniformity in TH distribution was evident in xeno‐free conditions (Fig. 2B, 2E; supplemental online Fig. 5D, 5I) and the proportion of floor‐plate neurons (denoted by TH/FOXA2 coexpression [25, 28]) was also significantly increased (>60%) (Fig. 2D–2G; supplemental online Fig. 5E, 5J). Robust TH and FOXA2 expression was similarly observed across additional hESC (HES3) and hiPSC (409B2) lines in xeno‐free conditions (supplemental online Fig. 3F–3J, 4F–4J). Furthermore, observation of stable FOXA2 expression between D11 and D25 indicates that superior fate specification under newly defined culture conditions is reflective of true gains rather than an accelerated differentiation trajectory (Fig. 2H).

Definitive vmDA neuronal identity can only be assigned following further narrowing of cell identity with markers such as EN1, nuclear orphan receptor NURR1 or paired‐like homeodomain transcription factor PITX3 [29]. At D25 in vitro, we demonstrate widespread coexpression of NURR1 with TH (Fig. 2I–2K). More definitive, however, is the rostro‐caudal and medial‐lateral expression of PITX3, which is highly enriched in the vm (Fig. 2M). Combined PITX3 expression, together with FOXA2 (Fig. 2L) and TH, unambiguously denotes vmDA neurons (Fig. 2N) and precludes misclassification with diencephalic or hindbrain TH^+^ populations. To this end we took advantage of our recently generated H9::PITX3‐eGFP knockin reporter line [19]. Differentiation revealed widespread PITX3 expression throughout xeno‐free cultures that robustly correlated with both TH and FOXA2 (Fig. 2P–2T). In all, 45.7% of total cells expressed PITX3 at D25, and strikingly 84.5% of TH^+^ cells were found to coexpress PITX3^+^ (Fig. 2O).

**Figure 3:**
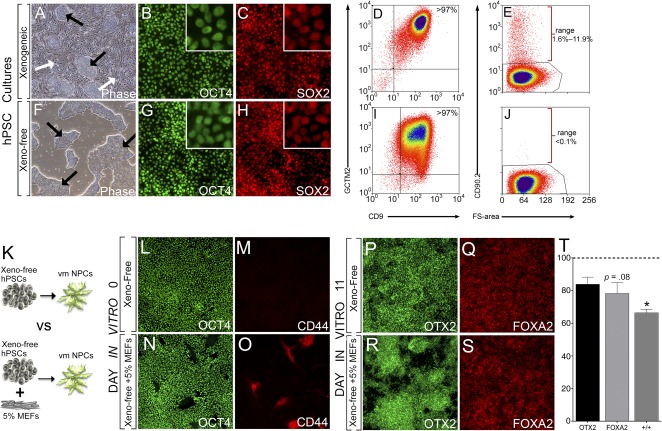
Residual fibroblasts, at the initiation of vmDA differentiation, influence fate specification. **(A, F):** Phase contrast image of hPSCs cultured in xenogeneic conditions on MEFs **(A)** or xeno‐free conditions on laminin‐521 **(F)**. Black arrows: hPSC colonies, white arrows: MEFs. Xenogeneic and xeno‐free hPSCs coexpressed pluripotency markers OCT4 **(B, G)** and SOX2 **(C, H)**. Analysis of pluripotent surface antigens CD9 and GCTM2 by FACS revealed that xenogeneic cultured hPSCs **(D)** were indistinguishable from xeno‐free cultures **(I)**. **(E, J):** Despite depletion, xenogeneic conditions retained a small fraction of CD90.2^+^ MEFs **(E)**, which were absent in xeno‐free conditions **(J)**. **(K):** Xeno‐free vmDA cultures were spiked at initiation with MEFs to elucidate potential effects on differentiation to 11 day ventral midbrain NPCs. **(L, M):** Xeno‐free cultures at DIV0 widely expressed OCT4 and were devoid of CD44^+^ fibroblasts. **(N, O):** The presence of fibroblasts demonstrated in xeno‐free DIV0 spike cultures confirmed by CD44 reactivity. Confirmation of vmDA precursor specification under xeno‐free conditions at DIV11 by immunolabeling for OTX2 **(P)** and FOXA2 **(Q)**. Cultures spiked with MEFS demonstrated reduced numbers of OTX2‐expressing **(R)** and FOXA2‐expressing **(S)** cells. **(T):** Percentage decrease in the proportion of cells in 11‐day vmDA cultures expressing OTX2, FOXA2, and OTX2/FOXA2 following spiking at D0 with MEFs, compared with standard xeno‐free cultures. Note there is a significant reduction in specification of OTX2/FOXA2 DA precursors following MEF spiking of the cultures. *n* = 3 culture replicates, data represent mean ± SEM. ∗, *p* < .05. Phase contrast images at ×40, immunofluorescence at ×100 magnification. Abbreviations: DA, dopamine; DIV, day in vitro; FACS, fluorescence‐activated cell sorting; hPSC, human pluripotent stem cell; NPC, neural progenitor cell; MEF, mouse embryonic fibroblast; vmDA, ventral midbrain dopaminergic.

### Identification of Mechanism Underlying Enhanced vmDA Specification in Xeno‐Free Cultures

In light of the improved differentiation outcomes under xeno‐free conditions, we sought to investigate the potential underlining mechanisms. Although it is speculated that fully defined media improves the homogeneity of cells before differentiation, we observed no differences in the pluripotency of hPSCs cultured in TeSR2 compared with MEF‐based xenogenic conditions, as assessed by OCT4 and SOX2 expression (Fig. 3A–3C, 3F–3H), as well as surface markers CD9 and GCTM2 (Fig. 3D, 3I, supplemental online Fig. 6I, 6J)—markers that have been shown to discriminate gradients of the pluripotency spectrum [30]. Instead, using the stromal cell marker CD90.2, a variable fraction of residual feeders were seen to remain within xenogeneic hPSC suspensions at the initiation of differentiation (1.6%–11.9% of the total live fraction, Fig. 3E; mean, 5.40% ± 3.28%) despite undergoing a widely utilized feeder depletion process [4, 31, 32]. No feeders were identified in xeno‐free hPSC preparations (Fig. 1J). To determine whether these contaminating cells underlie perturbed differentiation efficiencies xeno‐free cultures were “spiked” with 5% of feeders at the initiation of differentiation (Fig. 3K). CD44^+^ MEF cells could be identified within the culture at DIV0 (Fig. 3L–3O); however, most inactivated MEFs were lost/undetectable within 72 hours of differentiation, potentially being overgrown by expanding human NPCs (data not shown). Analysis at DIV11 revealed a 33.7% decrease in the proportion of mid‐forebrain floor plate OTX2+/FOXA2+ cells following MEF spiking, (Fig. 3P–3T), suggesting that the presence of contaminating MEFs elicits a destabilizing effect on early fate specification.

### Xeno‐Free vmDA Differentiation Is Amenable to Scalability and Cryopreservation of Progenitors

**Figure 4:**
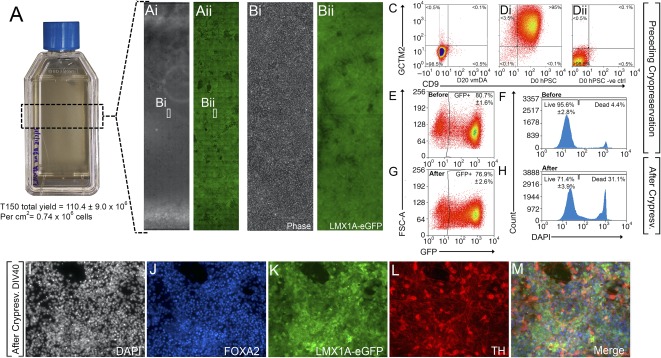
Scaled differentiation and cryopreservation of xeno‐free vmDA precursors. **(A, B):** Xeno‐free differentiation in flasks yields high cell quantities enriched for LMX1A‐eGFP at D22. **(C, D):** Scaled vmDA cultures **(C)** did not include pluripotent GCTM2/CD9 cells compared with pluripotent controls **(Di)** and unstained controls **(Dii)**. **(E, F):** Culture flask expansion had no impact on fate specification as revealed by LMX1A‐eGFP expression **(E)** or cell survival **(F)**. **(G, H):** Cryopreservation and thawing also had no impact on LMX1A‐eGFP expression **(G)** and minimal impact on survival **(H)**. After thawing, LMX1A‐eGFP vmDA progenitors maintained their capacity to differentiate into DA neurons, as revealed by DAPI **(I)**, FOXA2 **(J)**, LMX1A **(K)**, TH **(L)**, and merged **(M)** expression at D40. *n* = 3 culture replicates, mean ± SEM. Phase contrast images are at ×40, immunofluorescence at ×200 magnification. Abbreviations: D, day; DA, dopamine; DAPI, 4′,6‐diamidino‐2‐phenylindole; GFP, green fluorescent protein; hPSC, human pluripotent stem cell; TH, tyrosine hydroxylase; vmDA, ventral midbrain dopamine.

Necessary for the clinical applicability of defined differentiation protocols is the requirement for scalability. In this study, we demonstrate the ability to significantly scale‐up vmDA differentiation by 450‐fold (initiating differentiation in T150 flasks by comparison with previous results produced in 96‐well plates). The increased scale had no obvious effect on the survival, morphology or expression of LMX1A‐eGFP in culture (Fig. 4A, 4B), compared with previously observed results (Figs. 1, 2). At D22, the total cell yield averaged 110.4 ± 9.0 × 10^6^ per T150 flask (Fig. 4A), with 80.7% ± 1.6% of live cells expressing eGFP (Fig. 4E, 4F). vmDA NPCs were screened for the presence of pluripotent cells to assess teratoma formation risk following large‐scale expansion. No GCTM2^+^/CD9^+^ coexpressing cells were identified (Fig. 4C–4Dii).

In addition to the necessity for scalability for clinical translation, we examined the feasibility of cryopreserving vmDA precursors at a stage in the differentiation likely to be amenable to cell transplantation. Such approaches could enable large‐scale standardized differentiation and storage for “on‐demand” needs as reported in other hPSC fields [25]. Scaled vmDA NPCs were cryopreserved at day 22 in a xeno‐free cryopreservation solution using a controlled rate freezer. Postthaw vmDA NPCs, frozen in liquid N2 for up to 14 days, demonstrated a high viability (>71%; Fig. 4H) and retained LMX1A‐eGFP expression (76.9 ± 2.6% postthaw vs. 80.7% prefreeze) (Fig. 4E, 4G). Furthermore, culture of resurrected vmDA progenitors to day 40 produced correctly patterned vmDA neurons coexpressing LMX1A‐eGFP, FOXA2, and TH (Fig. 4I–4M).

### Xeno‐Free Cultures Yield Increased Proportions of Functionally Mature DA Neurons

**Figure 5:**
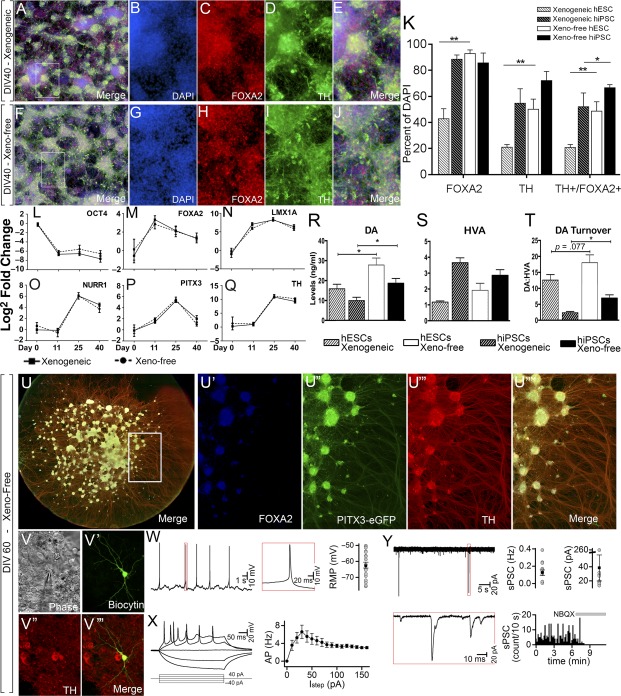
Maturation of hPSCs under xeno‐free conditions improved the yield of mature DA neurons and DA metabolism. Neurons displayed electrophysiological properties of classical vmDA neurons. Images of hPSCs differentiated under xenogeneic **(A–E)** or xeno‐free **(F‐J)** conditions illustrating the composition of DAPI, FOXA2, and TH labeled neurons. **(K):** Quantification of subpopulations revealed a significant increase in DA neurons under xeno‐free conditions across lines. **(L–Q):** Temporal gene expression profile of xenogeneic and xeno‐free cultures for OCT4 **(L)**, FOXA2 **(M)**, LMX1A **(N)**, NURR1 **(O)**, PITX3 **(P)**, and TH **(Q)**. **(R–T):** High‐performance liquid chromatography revealed a significant increase in dopamine release **(R)** and dopamine turnover **(T)** (DA:HVA ratio) in xeno‐free compared with xenogeneic cultures. **(S):** No change was observed in HVA levels. **(U):** Extended culture of H9::PITX3‐eGFP reporter vmDA neurons to D60 generated dense networks of DA neurons, as revealed by FOXA2/PITX3‐eGFP/TH coexpression, with extensive axonal fasciculation. **(V):** Cells were targeted for whole cell patch clamping. **(V′):** Biocytin‐labeled neuron coexpressing TH after recording. **(W):** Representative trace of action potential generation at resting membrane potential. **(X):** All cells exhibited inward rectification in response to hyperpolarization steps and generated action potentials. **(Y):** Trace recording of vmDA neuron receiving spontaneous excitatory postsynaptic currents, which could be blocked by administration of the AMPA antagonist, NBQX. *n* = 3 culture replicates (immunocytochemistry and high‐performance liquid chromatography), *n* = 6 culture replicates (qPCR), *n* = 21 recorded neurons (electrophysiology). Mean ± SEM. ∗, *p* < .05; ∗∗, *p* < .01; ∗∗∗, *p* < .001. Immunofluorescence, ×100 magnification; electrophysiology images, ×1,000 magnification. Abbreviations: AP, action potential; D, day; DA, dopamine; DAPI, 4′,6‐diamidino‐2‐phenylindole; DIV, day in vitro; hESC, human embryonic stem cell; hiPSC, human induced pluripotent stem cell; hPSC, human pluripotent stem cell; HVA, homovanillic acid; TH, tyrosine hydroxylase; vmDA, ventral midbrain dopamine.

Extended in vitro differentiation of vmDA progenitors (until D40) resulted in a significant increase in the proportions of TH^+^ neurons (hESC H9 = 50.1%, HES3 = 55.7%; hiPSC RM3.5 = 76.2%, of total cells) and cells coexpressing FOXA2^+^/TH^+^ (hESC H9 = 48.6%, HES3 = 52.9%; hiPSC RM3.5= 67.9%, of total cells; Fig. 5A–5K, supplemental online Fig. 7). Complementary to immunocytochemistry findings, temporal gene expression showed profiles indicative of vmDA fate specification for both xenogeneic and xeno‐free differentiation cultures, with the early downregulation of the pluripotency gene *OCT4*, progressive upregulation of vmDA progenitor/neuronal genes *FOXA2*, *LMX1A*, and *NURR1*, and subsequent elevation of mature markers *PITX3* and *TH* (Fig. 5L–5Q). Examination of single gene expression profiles did not reveal the striking differences in fate specification observed between xenogeneic and xeno‐free conditions, suggesting that the global measurements of mRNA levels in culture have limited capacity to distinguish differences in the numbers of individual cells coexpressing the multiple markers required to detect bona fide vm precursor and mature populations.

Critical for the function of DA neurons is their ability to synthesize and utilize dopamine. To this end, we assessed dopamine metabolism in KCl‐depolarized neurons using high‐performance liquid chromatography (HPLC). Reflective of increases in DA neurons observed at D40, dopamine levels were significantly elevated in both H9 hESC cultures differentiated under xeno‐free conditions compared with xenogeneic cultures (27.8 ± 3.5 and 15.9 ± 2.2 pmol/ml, respectively) as well as hiPSC RM3.5 cultures (18.6 ± 2.3 and 9.9 ± 1.7 pmol/ml, Fig. 5R). Although levels of the dopamine metabolite HVA remained unchanged (Fig. 5S), dopamine turnover (the ratio of dopamine to HVA, and an indicator of increased DA metabolism) was notably higher for xeno‐free differentiated hESC and significantly elevated for xeno‐free differentiated iPSC compared with xenogeneic conditions (6.9 ± 1.0 vs. 2.4 ± 0.4; Fig. 5T).

To further validate whether the hPSC‐derived vmDA cells generated under xeno‐free conditions were functional, we allowed them to mature to 55–65 days and performed whole cell patch clamping. At this stage of maturation the cells showed morphological features of mature DA neurons—with the majority expressing FOXA2, PITX3‐eGFP, and TH, in conjunction with extensive axonal growth (Fig. 5U), which was complemented by electrophysiological features indicative of vmDA neurons. Recorded cells (*n* = 21) exhibited a resting membrane potential of −62 ± mV, with 38% firing spontaneous action potentials of prolonged duration (Fig. 5W). All neurons generated action potentials upon depolarizing current steps and exhibited inward rectification in response to hyperpolarization steps (Fig. 5X). These findings are in agreement with previously published data from TH^+^ vmDA neurons [16, 22]. Several recorded cells exhibited sEPSCs (0.13 ± 0.03 Hz, 38 ± 18 pA) with fast kinetics that were blocked by administration of the AMPA antagonist NBQX (20 μM), whereas no cells exhibited outward inhibitory postsynaptic currents (Fig. 5Y). These findings indicate cells developed excitatory glutamatergic synapses, as previously described for DA neurons. Recorded neurons were confirmed as exhibiting a DA identity by colocalization of TH with biocytin (Fig. 5V). Combined, these findings suggest that xeno‐free cultured cells were capable of differentiating into functional DA neurons possessing morphological, gene, and protein expression and electrophysiological properties of endogenous midbrain DA neurons.

### Transplantation of Xeno‐Free DA Progenitors Results in Grafts With Improved Phenotype Composition and Restoration of Motor Deficits in Parkinsonian Rodents

**Figure 6:**
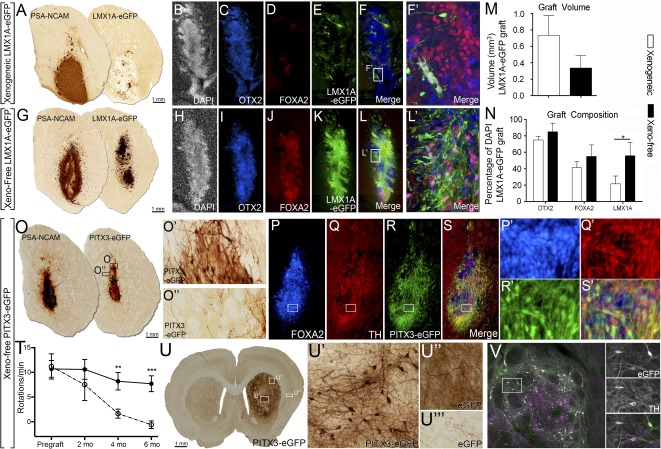
Transplantation of xeno‐free vmDA progenitors improved the yield of correctly specified cells within grafts of Parkinsonian mice. **(A, G):** Chromogenic PSA‐NCAM and GFP staining of transplanted H9::LMX1A‐eGFP progenitors differentiated for 25 days under xenogeneic **(A)** or xeno‐free **(G)** conditions and analyzed at 1 month after implantation. Note similarity in graft size, shown by PSA‐NCAM and enrichment of LMX1A‐eGFP cells under xeno‐free conditions. DAPI **(B, H)**, OTX2 **(C, I)**, FOXA2 **(D, J)**, LMX1A‐eGFP **(E, K)**, and merged images **(F, L)** show that xeno‐free derived grafts were enriched for DA progenitors, as shown by increased OTX2/FOXA2/LMX1A‐eGFP coexpression seen in the high‐power images: xeno‐free **(L′)** compared with xenogenic **(F′)**. **(M):** Volumetric analysis revealed no difference in graft size between xenogeneic or xeno‐free conditions. **(N):** Quantification of progenitors within the graft. **(O):** Chromogenic PSA‐NCAM and GFP staining of transplanted H9::PITX3‐eGFP progenitors, differentiated for 25 days under xeno‐free conditions and analyzed at 1 month after implantation. Grafts show high levels of PITX3‐GFP staining **(O, O′)** and modest GFP innervation of the host striatum **(O′′)**. Immunohistochemistry for FOXA2 **(P)**, TH **(Q)**, PITX3‐eGFP **(R)**, and merged **(S)** confirmed that a significant proportion of cells possessed a vmDA phenotype. **(P′, S′)**: Higher magnification images of **(P)–(S)**. Animals grafted with H9::PITX3‐eGFP progenitors (open circles) showed a significant improvement in amphetamine induced rotational asymmetry compared with 6‐OHDA lesioned controls (closed circles). **(U):** Representative H9::PITX3‐eGFP graft showing integration into the host striatum at 6 months. **(U′, U′′):** Grafts contained a dense network of cells and reinnervation of the dorso‐lateral striatum **(U′′)** as well as other vmDA targets, including the overlying cortex. LMX1A‐GFP cell grafts: *n* = 6 animals per group (xenogeneic and xeno‐free). PITX3‐GFP grafts: *n* = 3 at 1 month, and *n* = 6 at 6 months. *n* = 6 lesion controls. Mean ± SEM. ∗, *p* < .05. Abbreviations: DA, dopamine; DAPI, 4′,6‐diamidino‐2‐phenylindole; eGFP, enhanced green fluorescent protein; GFP, green fluorescent protein; TH, tyrosine hydroxylase; vmDA, ventral midbrain dopamine.

Next, we examined the capacity of the expanded vmDA progenitors, differentiated under xenogeneic or xeno‐free conditions, to survive intrastriatal transplantation in 6‐OHDA lesioned mice. The use of the H9::LMX1A‐eGFP H9 hESC reporter line enabled tracking of the grafted progenitors and assessment of their relative contribution to the graft. In all animals, healthy, viable grafts, confined to the striatum and showing no signs of tumor formation or neural overgrowth, were observed. Examination of hPSA‐NCAM staining, to identify human specific tissue in situ, highlighted no significant difference in graft size between xenogeneic and xeno‐free derived cell grafts, although considerably greater variation in graft size was observed in xenogeneic grafts (Fig. 6A, 6G, 6M: xenogeneic: 0.74 ± 0.24 mm^3^; xeno‐free: 0.38 ± 0.06 mm^3^). Examination of GFP staining however revealed that grafts derived from xeno‐free differentiation cultures contained notably more LMX1A^+^ progenitors than grafts of xenogeneic‐derived cells (Fig. 6A, 6G). These findings suggest that improved specification observed under xeno‐free conditions in vitro was maintained and influenced the fate of grafted cells in vivo. Further phenotype characterization of grafts at 1 month after implantation revealed high expression of OTX2 (xenogeneic: 75 ± 4.6%; xeno‐free: 85 ± 4.3%) and FOXA2 (41.3 ± 7.3% vs. 55.0 ± 5.7%), with a nonsignificant trend toward improved composition in xeno‐free‐derived grafts (Fig. 6C, 6D, 6I, 6J, 6N). Significant phenotypic differences were only revealed through the use of the LMX1A‐eGFP reporter, with a 2.5‐fold higher number of LMX1A‐GFP^+^ cells in xeno‐free compared with xenogeneic derived grafts (21.7 ± 9.4% vs. 55.8 ± 6.6%; Figure 6E, 6K, 6N).

To assess the long‐term integration of xeno‐free vmDA progenitors we transplanted H9::PITX3‐eGFP cells into 6‐OHDA lesioned athymic rats. Similar to H9::LMX1A‐eGFP grafts, at 1 month after transplantation, ectopic H9::PITX3‐eGFP striatal grafts maintained fate restriction with the majority of grafted cells coexpressing FOXA2/TH/PITX3‐GFP (Fig. 6P–6S). At this time grafts showed limited reinnervation of the host tissue (Fig. 6O). Assessment of motor function revealed a stable motor deficit in 6‐OHDA lesioned rats in response to amphetamine induced rotational testing, which was significantly corrected in PITX3‐GFP vm progenitor grafted animals by 4 months (Fig. 6 T). Postmortem assessment of the grafts at 6 months revealed a dense network of GFP cells within the graft core (Fig. 6U’) and extensive reinnervation of the striatum (Fig. 6U), most notably in the dorsolateral striatum (Fig. 6U”, the vmDA target necessary for motor function). Additional innervation was also seen within the overlying cortex (Fig. 6U’”). Cells at 6 months maintained a vmDA regional identity with all GFP^+^ graft derived cells coexpressing TH (Fig. 6V).

## Discussion

In this study, we report a fully defined xeno‐free protocol for the generation of vmDA progenitors and mature vmDA neurons from hPSCs. Although the inconsistent nature of feeder coculture is recognized [15], studies have not previously been performed to directly assess their effects on neural differentiation outcomes. Our findings suggest the carry‐forward of a small proportion of MEF cells underlies some of the variability observed during differentiation. Through the removal of feeders and xenogeneic reagents we found a marked improvement in homogeneity and reproducibility of differentiation outcomes across multiple human ESC and iPSC lines. We report a significant increase in the yield of appropriately specified OTX2/FOXA2 progenitors and FOXA2/TH DA neurons, compared with previous reports [3, 4, 5, 6, 12]. Further, we demonstrate elevated DA metabolism and show functional electrophysiological properties reflective of mature vmDA neurons. The use of LMX1A and PITX3 reporter lines enabled further validation of correct vmDA progenitor (LMX1A/FOXA2/OTX2) and neuronal identity (PITX3/FOXA2/TH) of the resultant differentiated cells, at a level of detail not previously described. Of relevance, widespread use of broad RNA quantification techniques have been depended on to attribute subtype identities to differentiated neural cultures. In this study, we show considerable discrepancy between RNA and protein based quantification assays, as reported in other systems [33, 34]. Specifically, qPCR analysis of key identifier genes failed to separate xenogeneic and xeno‐free cultures, despite robust separation using immunocytochemistry based counting strategies. As mRNA levels reflect global gene expression, individual cell expression variation may confound RNA assessment methods. Furthermore, qPCR cannot discern colocalization of the multiple markers required to detect bona fide vmDA precursors and mature neurons. These findings highlight the necessity for stringent cytochemical analysis.

Recent studies have highlighted the functional integration of vmDA progenitors, but have predominantly relied on human specific antibodies such as human nuclear antigen, PSA‐NCAM or constitutively active fluorescent proteins to assess size and integration [5, 7, 8, 12], with limited assessments of graft composition. We assessed graft composition and integration using novel LMX1A‐eGFP and PITX3‐eGFP reporter lines enabling a specific assessment of the DA contribution to the graft. We demonstrated that xeno‐free derived grafts contain significantly more correctly specified vmDA progenitors (FOXA2/OTX2/LMX1A‐eGFP) than xenogeneic‐derived cell grafts of a similar size, as well as correctly specified mature DA neurons (FOXA2/PITX3‐eGFP/TH). Long‐term assessment of the grafts showed maintained specification and a dense network of PITX3‐GFP vmDA fibers throughout the denervated striatum, thereby providing the first report specifically of the dopaminergic contribution to reinnervate the host, above that previously reported [5, 7, 8, 12]. This dense network of vmDA neurons and fibers were derived from a clinically translatable protocol and were capable of correcting motor asymmetry in 6‐OHDA lesioned rats. The enhanced homogeneity and progenitor yield achieved within the present study, both in vitro and in vivo are of critical importance in the context of cell‐based therapy where undifferentiated cells or incorrectly specified neuronal populations can result in deleterious clinical outcomes.

Finally, we report the capacity for scalability using our xeno‐free, defined differentiation protocol, as well as the ability to cryopreserve vmDA progenitors at a stage amenable to transplantation. Scalability from 96‐well plates to flasks facilitates utility for a variety of purposes from in vitro disease modeling and drug screening to clinical transplantation trials. Specifically, expansion in T150 flasks resulted in roughly 110 million cells/flask, of which >80% are correctly specified (LMX1A/FOXA2/OTX2) at the transplantation window. The significance of these findings for clinical purposes can be fully realized in the context of preceding clinical trials using fetal tissue where as few as 100,000 surviving DA neurons were sufficient for significant reinnervation of the caudate putamen, F‐DOPA uptake and functional improvements [35, 36, 37, 38]. Furthermore, these cells are amenable to cryopreservation, with no significant impact on viability, culture progenitor composition or terminal differentiation potential, thereby presenting an “on demand” cell source for clinical application.

## Conclusion

Our findings address a number of critical stepping‐stones in the advancement of hPSCs for clinical applications. This readily scalable, cyropreservable, and defined xeno‐free protocol, primed for clinical translation, provides a highly efficient and standardized system that has been acutely interrogated with novel LMX1A‐ and PITX3‐eGFP reporter lines. These findings are highly relevant to a wide variety of downstream applications, from in vitro disease modeling and drug screening, to the rapidly approaching aspiration of cell replacement therapy for patients with Parkinson’s disease.

## Author Contributions

J.C.N. and C.W.G.: conception and design, provision of study material and/or participants, collection and/or assembly of data, data analysis and interpretation, manuscript writing, final approval of manuscript; W.F.A.: collection and/or assembly of data, data analysis and interpretation, final approval of manuscript; S.J.M. and C.R.B.: collection and/or assembly of data, data analysis and interpretation, manuscript writing, final approval of manuscript; A.G.E. and E.G.S.: conception and design, provision of study material and/or participants, manuscript writing, final approval of manuscript; J.M.H. and C.W.P.: provision of study material and/or participants, final approval of manuscript; L.H.T.: conception and design, manuscript writing, final approval of manuscript; C.L.P.: conception and design, financial support, provision of study material and/or participants, collection and/or assembly of data, data analysis and interpretation, manuscript writing, final approval of manuscript.

## Disclosure of Potential Conflicts of Interest

C.W.P. is a consultant for iRiccorgPharm Pty Ltd. and has research funding from Capsugel. The other authors indicated no potential conflicts of interest.

## Supporting information

Supporting Information
