# Supplementary material for: Efficiently Specified Ventral Midbrain Dopamine Neurons from Human Pluripotent Stem Cells Under Xeno‐Free Conditions Restore Motor Deficits in Parkinsonian Rodents
Source: Stem Cells Transl Med. 2016 Oct 14;6(3):937–48. doi: 10.5966/sctm.2016-0073 (PMC5442782; doi:10.5966/sctm.2016-0073)
Supplement: Supplementary file 1 — Supporting Information [file SCT3-6-0937-s001.pdf]

**Supplemental Information – Parish and Niclis et al.**

## Supplemental Figures

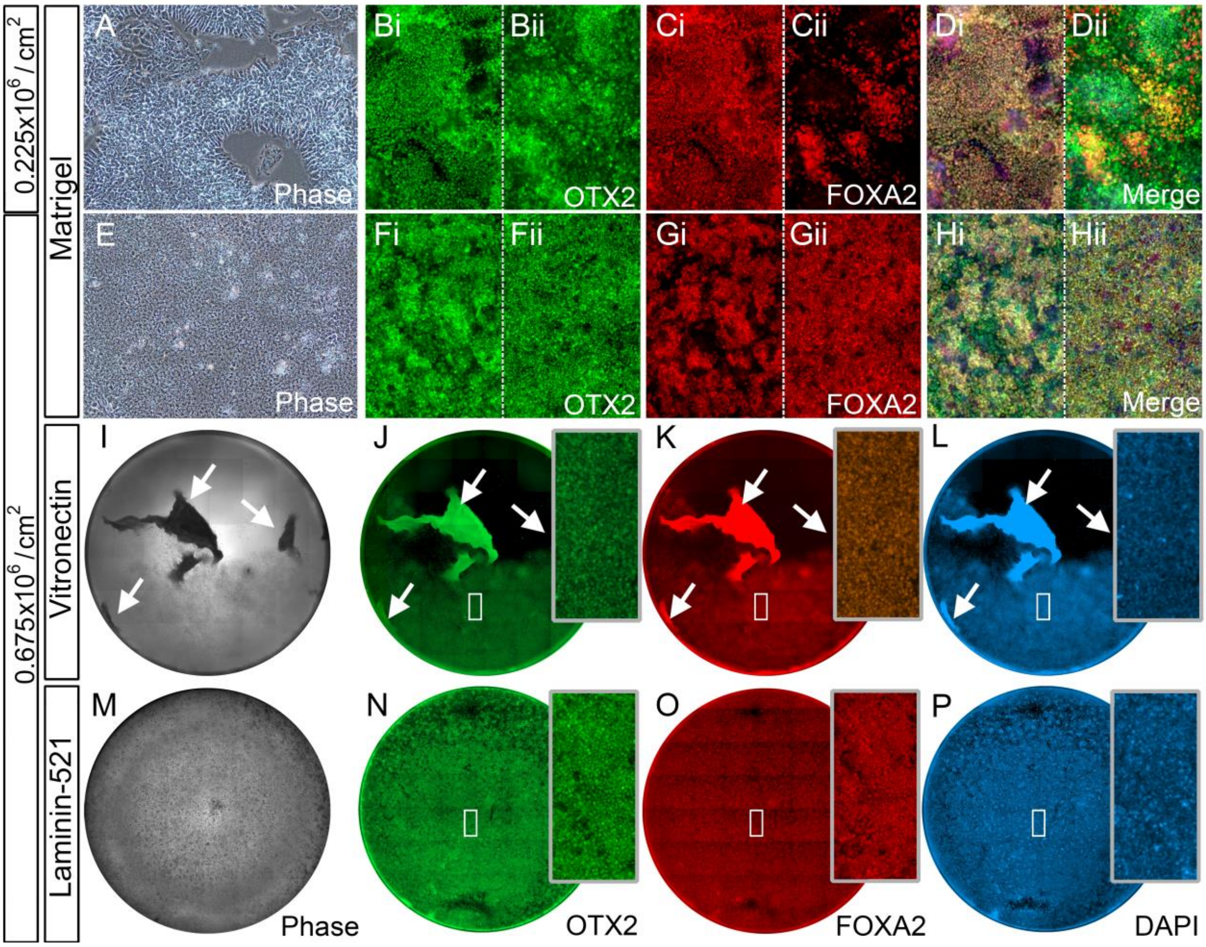

**Figure S1. Optimization of vmDA differentiation induction.** (A) Phase contrast of undifferentiated xenogeneic hPSCs prior to initiation of differentiation (day 0), with cells seeded at a standard literature density on MEFs ($0.225x10^6/cm^2$). Following 11 days of differentiation variable FOXA2 and OTX2 expression patterns were observed across independent cultures; (Bi-Di) robust patterning; (Bii-Dii) variable patterning. Increased xenogeneic hPSC seed densities on Matrigel ($0.675x10^6/cm^2$, E) improved differentiation reproducibility with robust expression of FOXA2 and OTX2 (Fi-Hi). Widespread OTX2 and FOXA2 expression was also observed from xeno-free hPSCs on Matrigel differentiated in fully defined and xeno-free media (Fii-Hii). Xeno-free hPSCs specification on xeno-free ECMs vitronectin (I-L) and recombinant human Laminin-521 (M-P), both of which exhibited robust OTX2 and FOXA2 expression. Vitronectin proved unreliable for long-term adherence of differentiated cultures (I-L, arrows indicating regions of cultures that have detatched) in comparison to rhLaminin-521. Immunofluorescence tiled 100x, phase contrast 20x magnification.

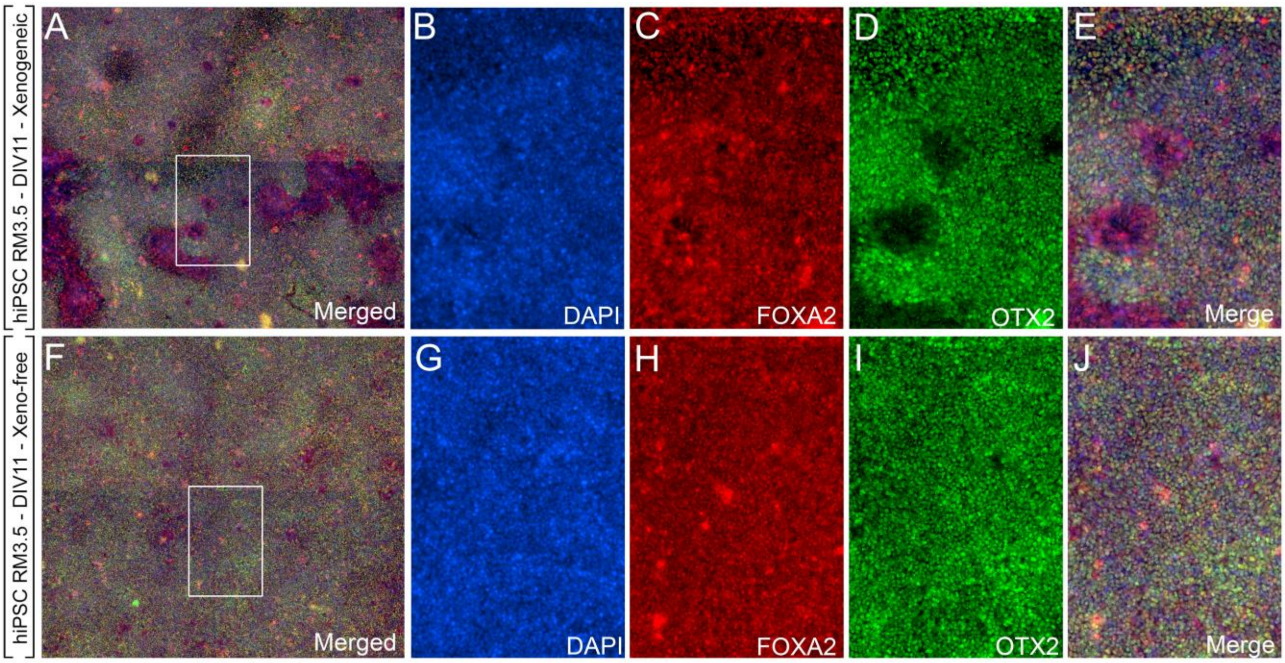


**Figure S2. Specification of vmDA precursors from hiPSC line RM3.5 under xenogeneic and xeno-free conditions.** Differentiation of hiPSC line, RM3.5, at 11 days *in vitro* under (A-E) xenogeneic and, (F-J) xeno-free conditions, illustrating DAPI, FOXA2, OTX2 and merged expression. Immunofluorescence tiled 100x magnification

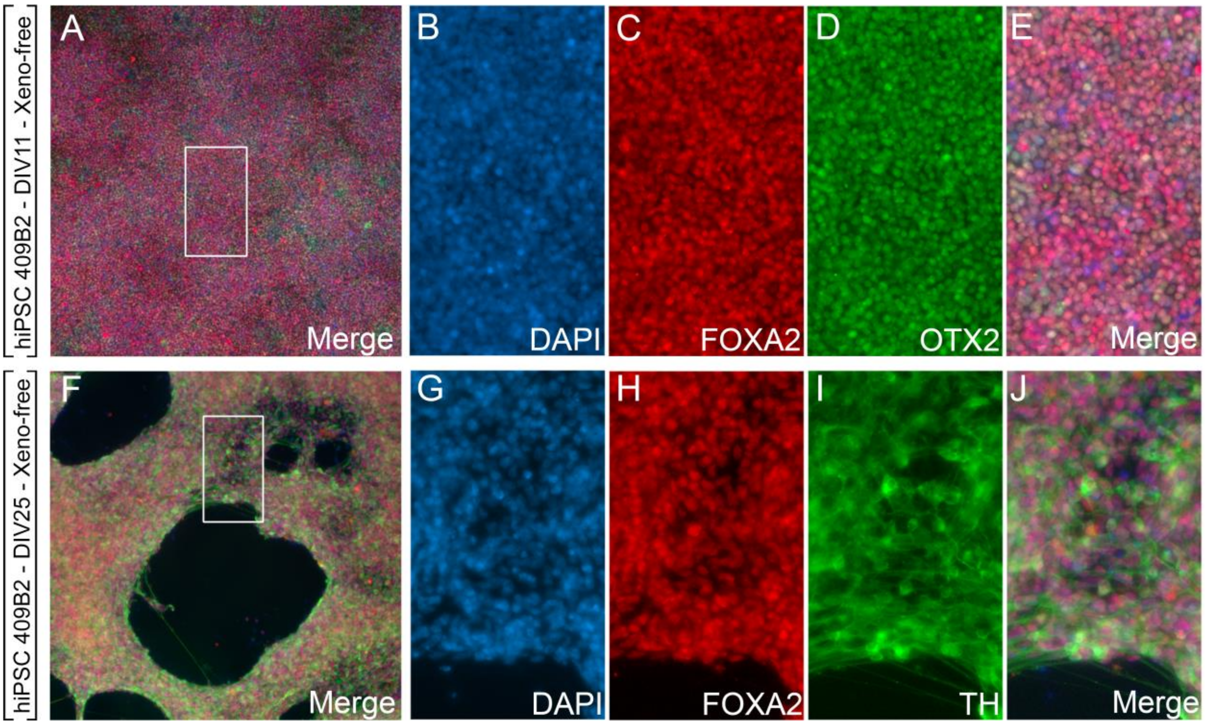


**Figure S3. Differentiation of hiPSC line 409B2 under xeno-free conditions.** Differentiation of hiPSC line 409B2 under xeno-free conditions resulted in high yields of vmDA precursors at day 11 in culture, as revealed by co-expression of DAPI, FOXA2, OTX2. High power image taken from panel (A) showing (B) DAPI, (C) FOXA2, (D) OTX2 and (E) merged. (F) Maintained differentiation (day 25 *in vitro*) resulted in specification of vmDA neurons, as confirmed by DAPI, FOXA2 and TH merged expression. High power image taken from panel (F) showing (G) DAPI, (H) FOXA2, (I) TH and (J) merged. Immunofluorescence tiled 100x magnification

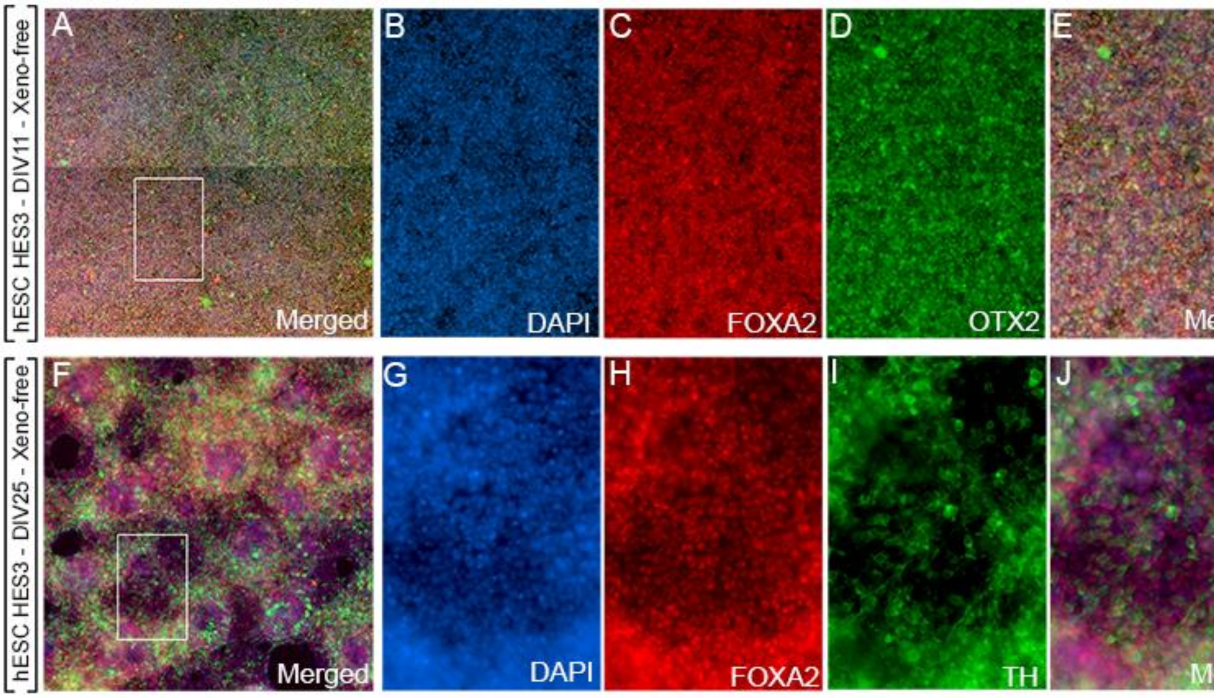


**Figure S4. Differentiation of hESC line HES3 under xeno-free conditions shows similar properties to the H9 hESC line.** (A) Differentiation of hESC line HES3 under xeno-free conditions resulted in high yields of vmDA precursors at day 11 *in vitro*, as revealed by co-expression of DAPI, FOXA2 and OTX2. High power image taken from panel (A) showing (B) DAPI, (C) FOXA2, (D) OTX2 and (E) merged. (F) Maintained differentiation (day 25 *in vitro*) resulted in specification of vmDA neurons, as confirmed by DAPI, FOXA2 and TH merged expression. High power image taken from panel (F) showing (G) DAPI, (H) FOXA2, (I) TH and (J) merged. Immunofluorescence tiled 100x magnification

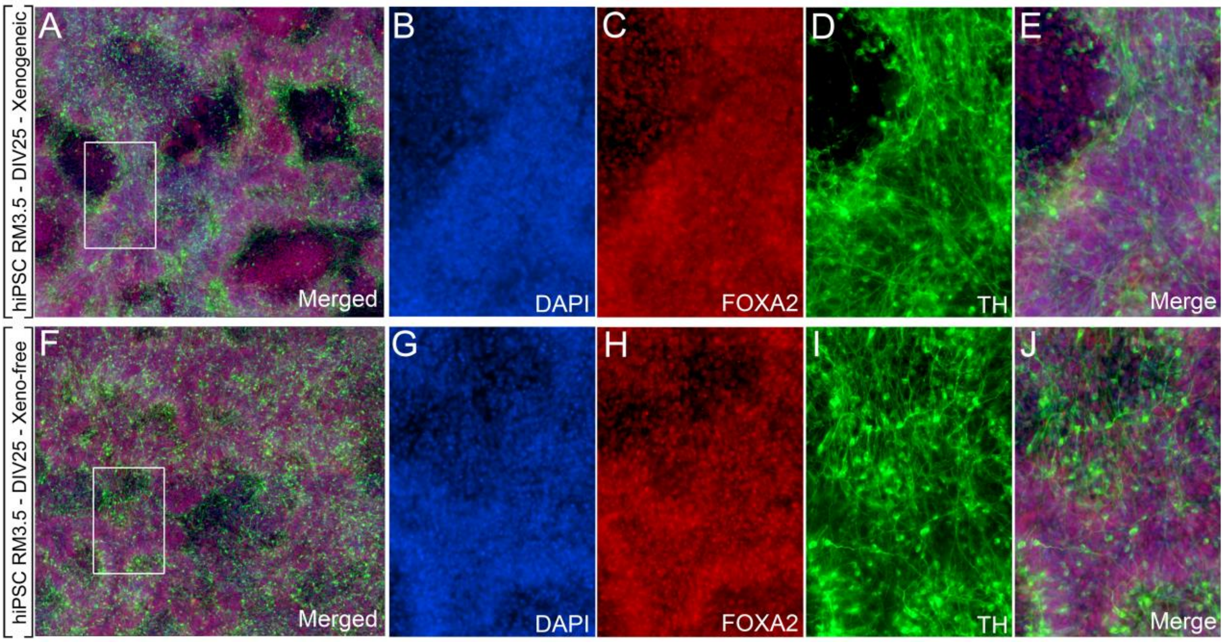


**Figure S5.** Differentiation of RM3.5 hiPSC line for 25 days in vitro under xenogeneic (A-E) and xeno-free conditions (F-J). (A,F) Representative merged images illustrating DAPI, FOXA2 and TH expression. High power image taken from panel (A – xenogeneic and F – xeno-free cultures) showing (B,G) DAPI, (C,H) FOXA2, (D,I) TH and (E,J) merge. Immunofluorescence tiled 100x magnification

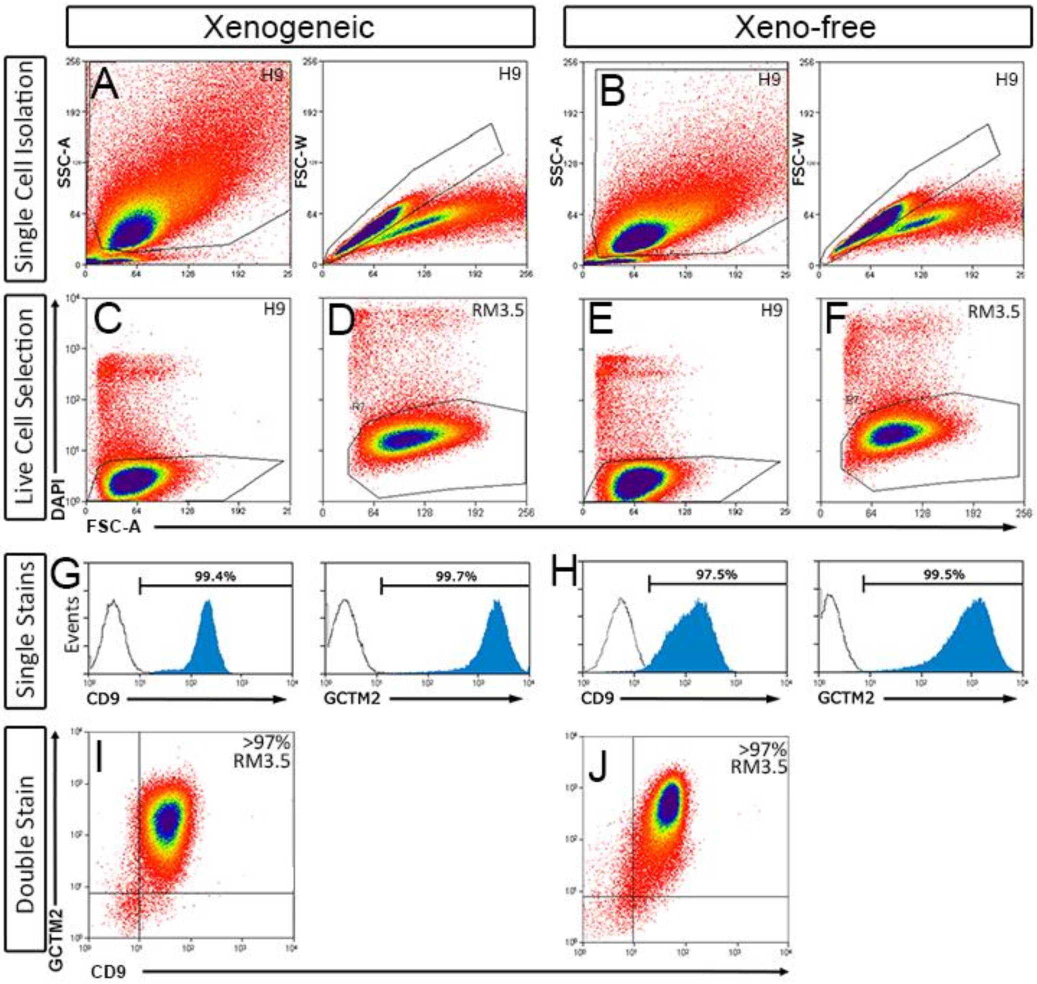


**Figure S6. Flow cytometry gating of pluripotent cells.** Gating strategy for undifferentiated xenogeneic (A,C,D) and xeno-free (B,E,F) hPSC cultures. Single stain controls delineate positive and negative populations (G-H). Representative images of double stained hiPSC populations in both xenogeneic (I) and xeno-free conditions (J).

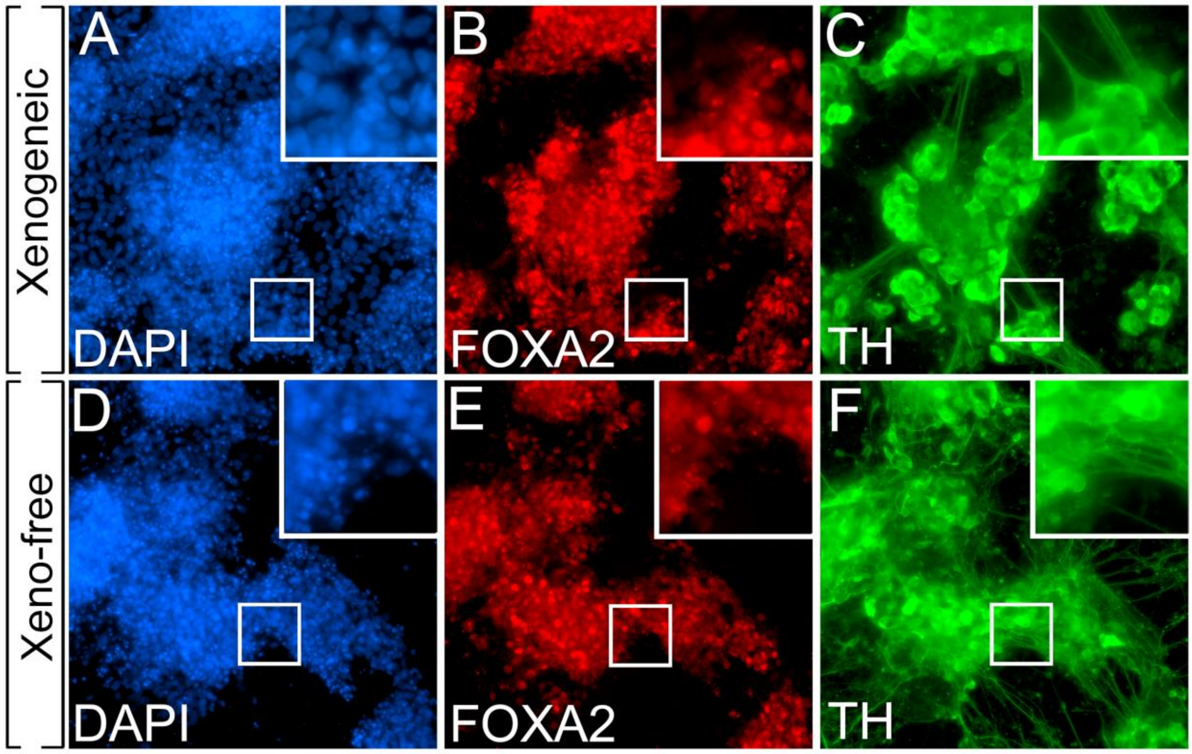


**Figure S7. Maturation of H9 hESC line under xenogeneic and xeno-free conditions.** (A) Representative merged images illustrating DAPI, FOXA2 and TH expression at day 40 in vitro under xenogeneic and (F) xeno-free conditions. High power image taken from panel (A and F) showing (B,G) DAPI, (C,H) FOXA2, (D,I) TH and (E,J) merge. Note the improved specification (FOXA2/TH co-localisation in image J) of hiPSC into DA neurons under xeno-free compared to xenogeneic conditions (E). Immunofluorescence tiled 200x magnification

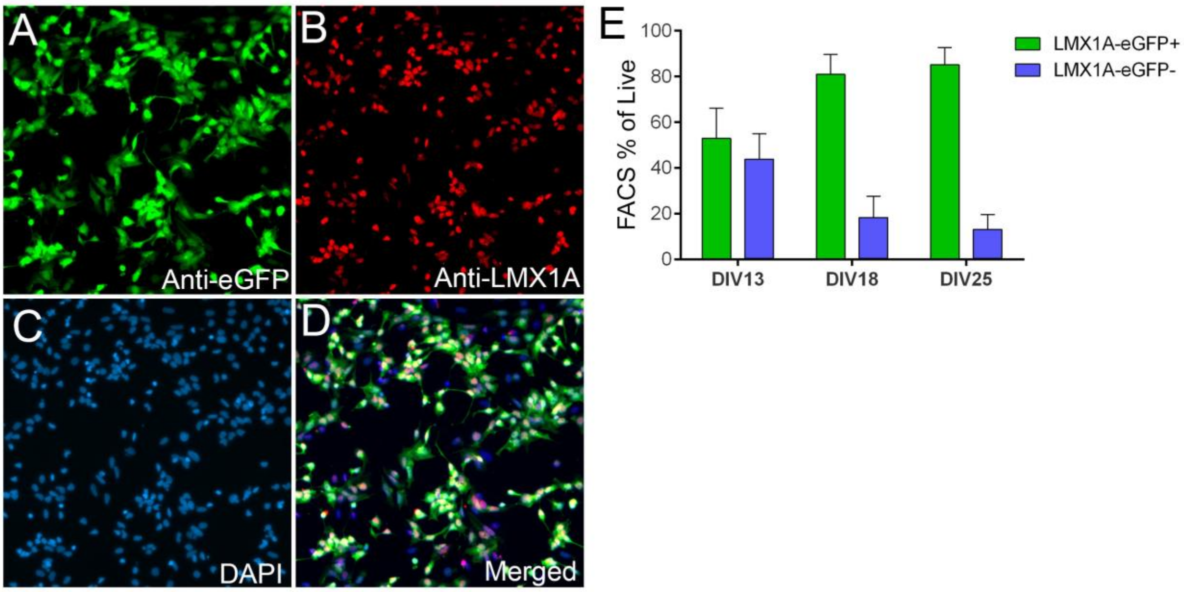


**Figure S8. LMX1A-eGFP reporter line fidelity.** (A) Representative images of vmDA NPCs stained for eGFP expression with an anti-GFP antibodies, (B) with anti-LMX1A/B antibodies (C) DAPI and (D) merged showing co-localisation between LMX1A/B expressing cells and eGFP expression. (E) Quantification of LMX1A-eGFP positive and negative live cell expression levels across a time-course of xeno-free differentiation, n=3 experimental replicates, mean±SEM. Immunofluorescence 200x magnification

**Supplemental Tables**

| Antibody Target | Species Raised In | Dilution | Conjugate | Company |
|---|---|---|---|---|
| OCT-4 | Mouse | 1:100 | n/a | SantaCruz |
| SOX2 | Goat | 1:100 | n/a | R&D Systems |
| GFP | Chicken | 1:1500 | n/a | Abcam |
| OTX2 | Rabbit | 1:300 | n/a | Millipore |
| FOXA2 | Goat | 1:300 | n/a | SantaCruz |
| NURR1 | Rabbit | 1:100 | n/a | SantaCruz |
| TH | Mouse; Rabbit | 1:800; 1:1000 | n/a | Millipore; Chemicon; |
| GFP | Rabbit | 1:20,000 | n/a | Abcam |
| CD9 IgG2A | Mouse | 1:100 | PE | Generous gift from Stem Cells Australia |
| GCTM2 IgM | Mouse | 1:100 | APC | |
| CD90.2 | Mouse | 1:50 | PE-Cy7 | |

**Table S1. Primary and secondary antibodies**

**Table S2. Primers used for qPCR analysis**

| Gene ID | Forward Primer (5'-3') | Reverse Primer (3'-5') |
|---|---|---|
| HPRT1 | CTTGCTGCGCCTCCGCCT | ATCACTAATCACGACGCCAGGGC |
| OCT4 | CTGGAGAAGGAGAAGCTGGA | CCTGTGTATATCCCAGGGTGA |
| FOXA2 | GCCCGAGGGCTACTCCTCCG | TCATGTTGCCCGAGCCGCTG |
| LMX1A | CTGCTGGGCAGAGCGGTGAG | TTTGCAGGAGGCGCACTGCA |
| NURR1 | TTTCTGCCTTCTCCTGCATT | CCCCCATTGTTGAAAGTCAC |
| PITX3 | CTGTCAGACGCTGGCACTC | TGCTTCTTTTTCAGCGAACC |
| TH | ACTGGTTCACGGTGGAGTTC | TCTCAGGCTCCTCAGACAGG |
